# Neoadjuvant and Adjuvant Systemic Therapies in Loco-Regional Treatments for Hepatocellular Carcinoma: Are We at the Dawn of a New Era?

**DOI:** 10.3390/cancers15112950

**Published:** 2023-05-27

**Authors:** Riccardo Nevola, Augusto Delle Femine, Valerio Rosato, Loreta Anesti Kondili, Maria Alfano, Davide Mastrocinque, Simona Imbriani, Pasquale Perillo, Domenico Beccia, Angela Villani, Rachele Ruocco, Livio Criscuolo, Marco La Montagna, Antonio Russo, Aldo Marrone, Ferdinando Carlo Sasso, Raffaele Marfella, Luca Rinaldi, Nicolino Esposito, Giuseppe Barberis, Ernesto Claar

**Affiliations:** 1 Liver Unit, Ospedale Evangelico Betania, 80147 Naples, Italy; valeriorosato@gmail.com (V.R.); pasqualeperillo@hotmail.it (P.P.); nicolinoe@gmail.com (N.E.); ernestoclaar@gmail.com (E.C.); 2 Department of Advanced Medical and Surgical Sciences, University of Campania “Luigi Vanvitelli”, 80138 Naples, Italy; augusto.dellefemine@studenti.unicampania.it (A.D.F.); maria.alfano@studenti.unicampania.it (M.A.); simo.imbriani@gmail.com (S.I.); domenico.beccia@studenti.unicampania.it (D.B.); angela.villani@studenti.unicampania.it (A.V.); rachele.ruocco@studenti.unicampania.it (R.R.); livio.criscuolo@unicampania.it (L.C.); m.lamontagna92@libero.it (M.L.M.); aldo.marrone@unicampania.it (A.M.); ferdinandocarlo.sasso@unicampania.it (F.C.S.); raffaele.marfella@unicampania.it (R.M.); luca.rinaldi@unicampania.it (L.R.); 3 Center for Global Health, Istituto Superiore di Sanità, 00161 Rome, Italy; loreta.kondili@iss.it; 4 Department of Mental Health and Public Medicine, University of Campania “Luigi Vanvitelli”, 80138 Naples, Italy; antonio.russo2@unicampania.it; 5 Medical Oncology, Ospedale Evangelico Betania, 80147 Naples, Italy; giuseppe.barberis59@libero.it

**Keywords:** hepatocellular carcinoma, recurrence, adjuvant therapy, neoadjuvant therapy, immunotherapy, immune checkpoint inhibitors

## Abstract

**Simple Summary:**

The treatment of hepatocellular carcinoma is burdened by an unacceptable recurrence rate after loco-regional treatment. Unlike all other solid tumors, no adjuvant or neoadjuvant systemic therapy schemes have been validated for hepatocellular carcinoma to date. Perioperative combination therapies could allow to optimize the radicality of treatments, reduce the recurrence rate and improve overall survival. Furthermore, systemic treatment could be used as bridging therapy in patients with hepatocellular carcinoma scheduled for liver transplantation with the aim of reducing the risk of dropout due to disease progression. The advent of immunotherapy in the therapeutic strategies of hepatocellular carcinoma could mark the start of a new era, revolutionizing the current management dogmas.

**Abstract:**

Despite maximizing techniques and patient selection, liver resection and ablation for HCC are still associated with high rates of recurrence. To date, HCC is the only cancer with no proven adjuvant or neoadjuvant therapy used in association to potentially curative treatment. Perioperative combination treatments are urgently needed to reduce recurrence rates and improve overall survival. Immunotherapy has demonstrated encouraging results in the setting of adjuvant and neoadjuvant treatments for non-hepatic malignancies. Conclusive data are not yet available in the context of liver neoplasms. However, growing evidence suggests that immunotherapy, and in particular immune checkpoint inhibitors, could represent the cornerstone of an epochal change in the treatment of HCC, improving recurrence rates and overall survival through combination treatments. Furthermore, the identification of predictive biomarkers of treatment response could drive the management of HCC into the era of a precision medicine. The purpose of this review is to analyze the state of the art in the setting of adjuvant and neoadjuvant therapies for HCC in association with loco-regional treatments in patients not eligible for liver transplantation and to hypothesize future scenarios.

## 1. Introduction

Hepatocellular carcinoma (HCC) is the fifth neoplasm in the world and the fourth leading cause of death from cancer [1]. The main risk factor for HCC is liver cirrhosis, most frequently caused by chronic HBV infection in Asian and sub-Saharan African regions and chronic HCV infection and alcohol and metabolic syndrome in Western populations [1,2]. Treatment options for early stage HCC include potentially curative techniques such as liver transplantation (LT), liver resection (LR) and radiofrequency ablation (RFA) [3]. In addition to the potential progression of liver disease, long-term survival of post-treatment patients is often affected by high recurrence rates, reaching 40–70% within 5 years [4]. Early (within 2 years of treatment) and late (more than 2 years) recurrences show different mechanisms, risk factors and prognosis [5]. In particular, early recurrence is the result of occult intrahepatic metastases closely linked to tumor burden and represents about 70% of all recurrence cases, whereas late recurrence is generally linked to a de novo neoplasm, with risk factors related to the underlying liver disease (e.g., cirrhosis, persistence of causes of liver injury) [6]. Early recurrences are associated with significantly lower overall and disease-free survival rates than late recurrences [5,7]. In addition to the characteristics of the primary tumor and the patient’s comorbidities, the type of treatment also influences the risk of recurrence. If LT offers the lowest recurrence rates (between 12% and 20% of cases) [8], in patients not candidates for transplantation, loco-regional treatments are burdened instead by high recurrence rates equal to about 25–50% for LR and up to 70% for RFA [5,7,9,10]. As for other malignancies, systemic pre- (neoadjuvant) or post- (adjuvant) loco-regional treatment could improve outcomes and significantly reduce recurrence rates. The advent of immunotherapy in HCC treatment has significantly improved the rate of response to systemic therapy and could offer the rationale for adjuvant and/or neoadjuvant therapeutic schemes also in the management of liver neoplasms.

The purpose of this review is to analyze the evidence currently available on the efficacy of neoadjuvant or adjuvant treatment regimens before or after loco-regional treatment for HCC in patients not eligible for LT hypothesizing their future perspectives.

## 2. Loco-Regional Treatments for HCC

Several determinants affect the decision-making process of choosing the most suitable treatment for HCC. In particular, the staging of the tumor (by Barcelona Clinic Liver Cancer—BCLC—staging system), residual liver function, recurrence predictors as well as age of the patient, performance status and presence of comorbidities are the factors that most influence the therapeutic choice. To date, the treatment options for HCC are multiple, ranging from potentially curative therapies (LT, LR, RFA) to palliative treatments (e.g., transarterial chemoembolization—TACE; transarterial radioembolization—TARE; systemic therapies) or supportive treatments (best supportive care—BSC) [3,11]. Furthermore, some treatments (such as RFA, TACE or TARE) allow the downstaging of the tumor and the passage from beyond to within the criteria for transplantability [12,13]. In addition to different indications and side effects, the various treatment techniques are characterized by different recurrence rates, which significantly impact overall survival (OS).

Liver transplantation (LT) represents the most radical therapeutic option currently available, able to treat both the neoplasm and the underlying liver pathology at the same time. It is indicated as the first-line therapy in patients with HCC who meet the Milan criteria (single tumor ≤ 5 cm or multiple tumors as ≤3 nodules size ≤ 3 cm without vascular and/or extrahepatic involvement) but are not eligible for surgical resection [3,11]. Furthermore, it can be reconsidered in patients previously excluded if a downstaging treatment (loco-regional or systemic) allows to meet the aforementioned criteria [12,13].

In patients who are not eligible for LT, the remaining therapeutic options consist of loco-regional (LR, RFA, TACE) or systemic treatments. Liver resection (LR) is the treatment of choice for single HCC of any size, without vascular invasion and/or metastasis, in non-cirrhotic or cirrhotic patients with preserved liver function and adequate predicted residual liver volume [3]. Despite a high OS (5-year survival between 40% and 70% in patients with very early HCC) [14], the overall risk of recurrence is between 25% and 50% of cases, with a higher risk for early rather than late recurrence [7,9,15]. Radiofrequency (RFA) or microwaves (MWA) thermal ablation are indicated in patients with early and very early HCC not suitable for surgery (transplant or resection) or as an alternative to LR in very early HCC stage with favorable localization [3]. The recurrence rates of patients treated with RFA appear significantly higher than those treated with LR [10,16]. Overall, the recurrence rate of HCC 5 years after treatment with RFA is approximately 70% [17], with trend proportional to the tumor burden (21.8% for unifocal HCC, 61.9% for bifocal HCC and 68.8% for trifocal HCC) [18]. Although it cannot be considered a radical treatment, transarterial chemoembolization (TACE) is currently one of the most frequently used therapeutic options for unresectable HCC. Since HCC shows an intense neoangiogenic activity, the intra-arterial administration of doxorubicin or platinum derivatives and the subsequent embolization of the peritumoral vessels are able to induce a cytotoxic and ischemic effect of the neoplastic mass. Compared to BSC, this technique offers significantly higher survival rates, equal to 51.8% at two years and 32.4% at five years after treatment [19].

## 3. Systemic Therapies for HCC

Conventionally, systemic therapies are reserved for advanced stage HCC not suitable for loco-regional treatment (e.g., vascular invasion and/or extrahepatic spread) or in case of progression in patients with preserved liver function [3,20]. Treatment regimens include the use of immune checkpoint inhibitors (ICIs, e.g., atezolizumab/bevacizumab) or tyrosine kinase inhibitors (TKIs, e.g., sorafenib, lenvatinib) as first-line or alternative therapeutic regimens (e.g., regorafenib, cabozantinib, ramucirumab) as second-line [21].

For many years, sorafenib has been the only approved drug for the treatment of advanced HCC. Sorafenib is an orally administered multikinase inhibitor able to suppress tumor growth through inhibition of serine/threonine kinases and blockade of the RAF/MEK/ERK pathway and to prevent tumor angiogenesis through inhibition of VEGF 1–3, PDF-β, FMS-like tyrosin kinase 3, serine/threonine kinase (c-RAF and b-RAF) and EGFR, suppressing tumor growth and metastasis [22]. Therefore, sorafenib has antiproliferative, antiangiogenic and proapoptotic properties and has been shown to significantly increase OS of HCC patients compared to placebo (10.7 vs. 7.9 months; *p* < 0.001) [23,24]. To date, it is indicated as a systemic therapy for HCC in patients with good liver function (Child-Pugh A) and advanced stage tumors (BCLC-C) or tumor progression after loco-regional treatments [3,11]. Lenvatinib was the first drug to demonstrate non-inferiority to sorafenib in the treatment of advanced HCC. As the latter, lenvatinib is also a multitarget TKI capable of inhibiting, inter alia, the receptor for FGF, VEGF and PDGF [25]. Treatment with lenvatinib is associated with an OS non-inferior to patients treated with sorafenib, but with a significantly greater response rate and progression-free survival compared to it (7.4 vs. 3.7 months) [26]. Lenvatinib is currently indicated as an alternative to sorafenib in patients with advanced stage HCC and preserved liver function [11].

In addition to TKIs, other drugs have gained approval for the treatment of advanced HCC in recent years. Immunotherapy has also shown promising results in this setting through the stimulation or modulation of the immune system against neoplastic cells. It includes immunomodulators (cytokines, chemokines), immune checkpoint inhibitors (ICIs), adoptive immunotherapy (lymphokine-activated killer—LAK-cells, cytokine-induced killer—CIK-cells, natural killer—NK-cells) and tumor vaccines [27]. The most promising results in the treatment of advanced HCC have been obtained with ICIs, which target programmed cell death (PD) receptor on T-cells or its ligands (programmed death ligand 1 and 2, PD-L1 and PD-L2) on cancer cells. ICIs are able to suppress antitumor immunity through inhibition of effector T-cell response by binding to the PD-1 receptor, PD-L1, expressed by tumor cells and/or tumor-infiltrating immune cells. Inhibition of the interaction between PD-L1 and PD-1 (e.g., by the monoclonal antibody atezolizumab) activates tumor-specific T-cell immunity. Through these mechanisms, ICIs have been shown to stimulate immune surveillance, resulting in a significant antitumor effect in several solid tumors including HCC [28].

Despite a response rate between 15% and 20%, monotherapy with PD-1 inhibitors does not result in a significant improvement in OS over standard of care, suggesting that the only inhibition of PD-L1/PD-1 axis may not be enough to determine adequate levels of antitumor immunity in the HCC setting [29,30]. However, by reducing VEGF-induced immunosuppression, anti-VEGF therapies are able to improve the efficacy of anti-PD-1 and anti-PD-L1 treatments [31,32]. For example, lenvatinib is able to reduce tumor PD-L1 levels and improve the efficacy of anti-PD-1 drugs through the inhibition of FGF receptor 4 (FGFR4) [33]. In order to increase its efficacy, anti-PD-L1 and anti-VEGF were therefore combined. The combination of atezolizumab (PD-L1 inhibitor) and bevacizumab (anti-VEGF monoclonal antibody) has been shown to determine significantly higher OS and progression-free survival rates than sorafenib [34,35,36]. In particular, 1-year OS was 67.2% with atezolizumab/bevacizumab and 54.6% with sorafenib whereas median progression-free survival was 6.8 months and 4.3 months, respectively [34]. Currently, this combination is indicated as first-line treatment in patients with locally advanced or metastatic unresectable HCC and preserved liver function [13]. However, patients with Child Pugh class (CP)-B recently reported similar tolerability compared to CP-A [37]. Other combinations of immunotherapy plus anti-angiogenic TKIs (e.g., cabozantinib-nivolumab, cabozantinib-atezolizumab, lenvatinib-pembrolizumab, camrelizumab-rivoceranib, nivolumab-ipilimumab) have been tested or are under evaluation with conflicting results [38,39,40,41]. Recently, the combination tremelimumab (a cytotoxic T lymphocyte-associated antigen-4 blocking antibody) plus durvalumab (anti PD-L1 monoclonal antibody) has been approved in the USA for the treatment of adult patients with unresectable HCC [42]. In the HIMALAYA phase III trial, this combination achieved a significantly longer OS than treatment with sorafenib alone [43], so it could be considered as a potential alternative to the atezolizumab/bevacizumab combination as first-line therapy of advanced HCC [11].

## 4. New Scenarios of Systemic Therapy for HCC

The efficacy of new therapeutic regimens has imposed a re-evaluation of the role of systemic therapy among the therapeutic options of HCC and opened new scenarios, such as the opportunity of sequential treatments [25]. In fact, the use of systemic therapy in combination with other techniques could optimize the outcomes and lower the currently high recurrence rates. In patients with HCC scheduled for LT, systemic treatment can be used as bridging therapy with the aim of reducing the risk of dropout due to disease progression, currently estimated at 25% at 1 year [44]. In patients not eligible for LT, on the other hand, adjuvant or neoadjuvant systemic therapies could reduce recurrence rates after loco-regional therapy, potentially increasing OS, similar to what has already been validated for non-hepatic neoplasms. Indeed, pre- or post-operative antiproliferative and antiangiogenic therapies could be able to decrease the occult intra- and extrahepatic dissemination frequently associated with the risk of early recurrence. Previous studies could not demonstrate a clear efficacy of neoadjuvant or adjuvant treatments with sorafenib, although some evidence suggested that adjuvant therapy could increase recurrence-free survival (RFS) [45]. The availability of new therapeutic regimens that have proven to be more effective than sorafenib and potentially complementary to it requires a re-evaluation of the effectiveness and role of systemic therapies before or after loco-regional treatment. In this regard, growing evidence is currently available, and several trials are underway.

### 4.1. Neoadjuvant Systemic Therapy for HCC

Similar to what has been validated for other non-hepatic neoplasms, in patients with high tumor burden (i.e., single large HCC, tumors adjacent to major vascular structures requiring narrow-margin hepatectomy, unilobar multifocal disease), neoadjuvant therapy could allow the downstaging of the tumor, favoring safer and more radical LR. Furthermore, neoadjuvant systemic therapy schemes could reduce the risk of early recurrence after loco-regional treatment and provide useful information on tumor susceptibility to anticancer drugs [28]. Before the use of ICIs in the treatment of HCC, the low efficacy and poor tolerability profile of the available drugs did not favor the development of neoadjuvant therapy schemes. However, the availability of new molecules with greater efficacy and a good tolerability profile has re-opened the possibility of pre-operative systemic therapies also in the HCC setting.

The current evidence on the role of systemic neoadjuvant therapy before loco-regional therapy for HCC are illustrated in Table 1. Some phase I [46] and II [47,48,49,50,51] trials have confirmed the feasibility and safety of neoadjuvant regimens in the treatment of the HCC and opened the way for their clinical study and validation. Preliminary data provided by Ho et al. [46] showed that neoadjuvant systemic therapy with cabozantinib + nivolumab was feasible and could result in margin-negative resections. This single arm phase Ib study performed on 15 patients with locally advanced HCC showed in particular that 12 patients (80%) had undergone successful negative margin resection after neoadjuvant therapy, and 5 patients had major pathologic responses. Similarly, Marron et al. [47] preliminarily evaluated the efficacy of neoadjuvant cemiplimab (an anti-PD-1) in patients with resectable HCC. A total of 21 patients with stage Ib, II and IIIb HCC received two cycles of cemiplimab (350 mg intravenously) every 3 weeks before LR and an additional eight cycles after. Among the 21 patients who underwent successful resection, 20% had achieved >70% tumor necrosis, 15% had a partial response, and all other patients showed stable disease. Kaseb et al. [48] evaluated safety and tolerability of perioperative nivolumab with or without ipilimumab in 27 patients with resectable HCC. The authors highlighted that both therapeutic schemes were safe and feasible and that adverse events (more frequent for the nivolumab + ipilimumab combination therapy) did not delay the LR. Preliminary data also showed that estimated median progression-free survival was 9.4 months with nivolumab and 19.53 months with nivolumab plus ipilimumab. Finally, Zhu et al. [50] analyzed the safety and impact of the neoadjuvant TACE plus PD-1 inhibitor (camrelizumab or sintilimab) in patients with intermediate-stage HCC. Patients receiving this regimen demonstrated 1-year and 2-year OS rates of 100.0% and 76.4%, whereas the 1-year RFS rate was 86.6%. Successful downstaging has been reported in 70% of patients undergoing neoadjuvant therapy and is associated with more prolonged RFS than patients with failed downstaging.

However, case-control trials are needed to validate these treatment protocols and confirm their efficacy. In this regard, Wu et al. [52] recently evaluated retrospectively the outcomes of patients treated with hepatectomy alone versus patients treated with triple neoadjuvant therapy using the combination of lenvatinib, anti-PD-1 monoclonal antibodies and TACE before LR. OS and RFS were significantly longer in the triple therapy group than in the LR-alone group. After 12 and 24 months of follow-up from the LR, the neoadjuvant therapy group showed an OS rate of 100% and 85.7% and an RFS rate of 66.95% and 48.8%, respectively, whereas the LR-alone group showed a rate of OS of 73.7% and 48.7% and an RFS rate of 28.34% and 22.99%. Furthermore, triple therapy reduces the rate of major vascular invasion and increases the probability of margin-negative resections. In addition to the benefit in patients with resectable HCC, this triple neoadjuvant therapy has been shown to favor the downstaging of unresectable HCC and allow resection in more than half cases with high objective response rates [53].

In this setting, triple combination therapy proved to be superior to dual combination therapy (Lenvatinib plus TACE) [54]. Wu et al. [52,53] hypothesize that lenvatinib may suppress HCC angiogenesis and antitumor immunity, potentially increasing the antiproliferative effect of PD-1 antibodies (not able alone to obtain adequate levels of anticancer immunity by PD-L1/PD-1 axis blockade) and counteracting the undesired neoangiogenic action of TACE (per se capable of increasing the efficacy of PD-1 antibodies through the release of tumor-specific antigens). The combination therapy would therefore act on different immune and antiproliferative patterns with a complementary and synergistic action of the individual treatments, resulting in high tumor response rates and a significant improvement in OS. Although the study has several limitations (retrospective design, significant imbalance between groups, abstract availability only), Xia et al. [55] provide additional data to support the potential efficacy of neoadjuvant therapy in HCC. In particular, neoadiuvant ICI (camrelizumab) combined with anti-angiogenic targeted drug (apatinib) for resectable HCC can reduce the 1-year recurrence rate and improve the 1-year survival rate, especially for those with solitary tumor.

Currently there are several ongoing trials in this setting. Pinato et al. [56], for example, are evaluating safety and bioactivity of the neoadiuvant nivolumab/ipilimumab combination before LR in early-stage HCC (trial registration number: NCT03682276). Li et al. [57] are, instead, analyzing the safety and efficacy of the combination scheme lenvatinib plus sintilimab plus radiotherapy as neoadjuvant treatment regimen in patients with HCC and portal vein tumor thrombus (NCT05225116). Finally, there is an ongoing evaluation by Zhang et al. [58] of the efficacy of neoadjuvant tislelizumab with stereotactic body radiotherapy in patients with resectable HCC (NCT05185531).

**Table 1:** Current evidences on the role of neo-adjuvant systemic therapy after loco-regional treatment for HCC.

Ref.	Year	First Author	Sample Size	Study Typology	Neoadjuvant Treatment	Loco-Regional Therapy	Control Group	Results
[46]	2021	Ho	15 patients with locally advanced HCC	Single arm, phase Ib trial	Cabozantinib + Nivolumab	LR	NA	Neoadjuvant Cabozantinib + Nivolumab is feasible and can result in margin-negative resections
[51]	2021	Woei-A-Jin	24 patients with early/intermediate-stage HCC	Single-arm trial	Dovitinib	RFA or MWA, TACE ± RFA, LR, Radioembolization	NA	Neoadjuvant Dovitinib is associated with intratumoral blood flow reduction and modest antitumor responses
[47]	2022	Marron	21 patients with resectable HCC	Single-arm, open-label, phase II trial	Cemiplimab (neoadjuvant and adjuvant)	LR	NA	20% of treated patients had obtained tumoral necrosis > 70%; 15% had a partial response; all other patients showed stable disease
[48]	2022	Kaseb	27 patients with resectable HCC	Single-center, randomized, open-label, phase II trial	Perioperative Nivolumab vs. Nivolumab + Ipilimumab	LR	NA	Perioperative nivolumab and nivolumab + ipilimumab is safe and feasible in patients with resectable HCC
[49]	2022	Xia	18 patients with resectable HCC	Single-arm, open-label, phase II trial	Camrelizumab + Apatinib (neoadjuvant and adjuvant)	LR	NA	Perioperative camrelizumab plus apatinib show a promising efficacy and manageable toxicity in patients with resectable HCC
[50]	2022	Zhu	20 patients with intermediate-stage HCC	NS	PD-1 inhibitor (camrelizumab or sintilimab) + TACE	LR	NA	Neoadjuvant TACE plus PD-1 inhibitor determines a downstaging rate of 70% and an acceptable survival profile
[52]	2022	Wu	24 cases and 76 controls	Retrospective	Lenvatinib + PD-1 + TACE	LR	LR alone	Neoadjuvant triple therapy significantly increased both the OS and DFS rates in resectable HCC with high risk of recurrence, compared with surgery alone
[55]	2022	Xia	14 cases and 115 controls	Retrospective	Camrelizumab + Apatinib	LR	LR alone	Neoadjuvant camrelizumab plus apatinib for resectable HCC can reduce the 1-year recurrence rate and improve the 1-year survival rate, especially for those with solitary tumor

DFS: disease-free survival; HCC: hepatocellular carcinoma; LR: liver resection; MWA: microwave ablation; NA: not applicable; NS: not specified; OS: overall survival; PD-1: anti-programmed death 1 antibodies; RFA: radiofrequency ablation; TACE: transcatheter arterial chemoembolization.

### 4.2. Adjuvant Systemic Therapy for HCC

If the main goal of neoadjuvant treatment schemes is to improve the resectability of tumor, adjuvant therapy after loco-regional treatment for HCC aims to reduce the recurrence rate and increase overall and disease-free survival. The two types of treatment (loco-regional and systemic) could have complementary effects: on the one hand, loco-regional treatments reduce tumor burden and induce the release of tumor antigens and proinflammatory cytokines; on the other, VEGF and TKIs boost antitumor immunity.

The evidence currently available on the role of adjuvant systemic therapy after loco-regional treatment is illustrated in Table 2. As for neoadjuvant therapies, previously available drugs had not demonstrated clear efficacy even in adjuvant treatment protocols. The current data appear, in fact, conflicting, and for this reason, HCC is the only cancer with no proven adjuvant therapy after potentially curative treatment to date. 

#### 4.2.1. TKIs-Based Adjuvant Treatments

Some retrospective studies [59,60,61,62,63,64,65,66] or small clinical trials [67,68] have found that adjuvant therapy with sorafenib can reduce the postoperative recurrence rate and improve OS. In particular, Zhang et al. [59] retrospectively analyzed the efficacy of adjuvant treatment with sorafenib in 113 patients who underwent R0 LR for HCC with microvascular invasion (MVI) comparing them to data from 113 matched patients who undergone LR alone. In this study adjuvant treatment with sorafenib was associated with significantly higher OS and RFS than the LR alone group, with similar results for both early or very early HCC (BCLC 0-A) and intermediate HCC (BCLC B).

Conversely, one of the few randomized clinical trials (RCTs) in this setting showed opposite results. In the STORM trial, Bruix et al. [69] prospectively evaluated the efficacy of sorafenib in 1114 patients with HCC after a complete radiological response after LR (*n* = 900) or RFA (*n* = 214). No differences in median RFS between the two groups (33.3 months in the sorafenib group vs. 33.7 months in the placebo group, *p* = 0.26) were noted. Furthermore, sorafenib was associated with poor toxicity profile (28% of patients experiencing grade 3 or 4 adverse event in the sorafenib group vs. <1% of patients in the placebo group), with four treatment-related deaths. However, a recent meta-analysis conducted on 2655 patients from 13 studies seems to contradict what was previously found in the STORM trial [70]. Indeed, the combined results of the studies would indicate that adjuvant therapy with sorafenib after LR could significantly increase OS (hazard ratio, HR = 0.71, 95%: confidence interval, CI = 0.59–0.86) and RFS (HR = 0.68, 95% CI = 0.54–0.86) and reduce recurrence rates. However, the authors underline that further evidence is needed before reaching definitive conclusions.

Recently, Lin et al. [71] evaluated the benefit of the combination of TACE + TKIs (sorafenib, lenvatinib or apatinib) after curative LR in HCC patients at high risk of early recurrence. The combination of adjuvant TACE + TKIs after LR has been shown to significantly increase the 1-year and 2-year RFS rate (45.5% and 34.9%, respectively) compared to post-operative TACE alone (26.8% and 18.3%, respectively). Patients with HCC diameter ≥ 5 cm, number of lesions < 3, absence of vascular and/or biliary infiltration, capsule integrity and stage IIIB (according to the American Joint Committee on Cancer 8th staging system) could benefit more from adjuvant TACE plus TKI treatment. A phase IIb clinical trial is currently underway aiming to evaluate the efficacy of adjuvant donafenib combined with TACE in patients with HCC at a high risk of recurrence after LR [72].

**Table 2:** Current evidences on the role of adjuvant systemic therapy after loco-regional treatment for HCC.

	Ref.	Year	First Author	Sample Size	Study Typology	Loco-Regional Therapy	Adjuvant Treatment	Control Group	Results
TKIs	[67]	2014	Wang	31 HCC patients who had undergone curative LR	Open label, controlled, phase II trial	LR	Sorafenib	LR alone	Adjuvant Sorafenib therapy significantly prolong time to recurrence after LR
	[59]	2014	Zhang	78 HCC patients	Retrospective	LR	Sorafenib	LR alone	Sorafenib did not significantly prolong RFS and did not reduce recurrence rate but significantly prolonged OS
	[69]	2015	Bruix	1114 HCC patients with a complete response after LR or RFA	Double-blind, phase III trial	LR or RFA	Sorafenib	Placebo	No difference in median RFS between Sorafenib and placebo
	[60]	2016	Xia	34 patients with BCLC-C stage HCC	Retrospective	LR	Sorafenib	LR alone	Adjuvant Sorafenib therapy is associated with significantly lower recurrence rate
	[61]	2016	Li	34 BCLC stage C HCC patients with portal vein thrombus	Retrospective	LR	Sorafenib	LR alone	Adjuvant Sorafenib therapy is associated with significantly longer TTP and OS compared to LR alone
	[68]	2016	Antoniou	30 HCC patients	NS	LR	Sorafenib	LR alone	No clinical benefits from adjuvant Sorafenib therapy
	[62]	2017	Liao	42 patients with advanced HCC and at a high risk of recurrence	Retrospective	LR	Sorafenib	BSC	Sorafenib improves RFS, but not OS, in patients with advanced HCC who underwent LR.
	[63]	2017	Zhuang	81 patients with intermediate/advanced HCC	Retrospective	LR	Sorafenib	LR alone	OS is significantly longer in the surgery and Sorafenib group than in the surgery-only group. RFS does not differ significantly between the two groups
	[64]	2019	Zhang	226 HCC patients with MVI who underwent R0 LR	Retrospective (PSM analysis)	LR	Sorafenib	LR alone	Adjuvant Sorafenib is associated with significantly better survival outcomes than LR alone for HCC patients with MVI
	[65]	2019	Huang	49 HCC patients with MVI after curative LR	Retrospective	LR	Sorafenib	LR alone	Adjuvant Sorafenib therapy in HCC patients with MVI is associated to better OS e RFS than LR alone
	[66]	2019	Wang	209 HCC patients	Retrospective	LR	Sorafenib	LR alone	One-year survival rate is significantly higher with sorafenib than observed with control
	[70]	2021	Huang	2655 patients (from 13 studies)	Meta-analysis	LR	Sorafenib	LR alone or placebo	Adjuvant Sorafenib therapy after LR could prolong OS and RFS and reduce recurrence rates
	[71]	2022	Lin	199 HCC patients with a high risk of early recurrence after LR	Multicenter retrospective	LR	TACE + TKI	TACE	TACE plus TKI treatment can reduce the incidence of early recurrence with tolerable adverse events
Tumor vaccine	[73]	2004	Kuang	41 HCC patients	Randomized phase II trial	LR	Autologous tumor vaccine	LR alone	Adjuvant autologous formalin-fixed tumor vaccine is a safe, feasible, and effective treatment for preventing postsurgical recurrence of HCC
	[74]	2014	Shimizu	94 patients with invasive HCC	Non-randomized phase II trial	LR	DCs vaccine + ATVAC	LR alone	Adjuvant DCs vaccine plus ATVAC significantly improve RFS and OS compared with LR alone
	[75]	2017	Lee	156 HCC patients	Multicenter phase II RCT	LR or RFA	Autologous DCs	LR or RFA alone	DCs immunotherapy significantly reduced the risk of recurrence of non-RFA group. Baseline serum IL-15 was correlated with RFS prolongation
AIT	[76]	2000	Takayama	150 HCC patients	RCT	LR	Autologous lymphocytes	LR alone	Adjuvant adoptive immunotherapy decreased the recurrence rate by 18% e significantly prolonged the time to first recurrence. No impact on OS
	[77]	2009	Hui	127 HCC patients	RCT	LR	CIK cells	LR alone	Adjuvant CIK cells therapy may prevent recurrence/metastasis after LR. No impact on OS
	[78]	2015	Xu	200 HCC patients	Single center RCT	LR	CIK cells	LR alone	Adjuvant CIK therapy prolong the TTR, but the treatment did not improve the RFS and OS
	[79]	2015	Lee	230 HCC patients	Multicenter RCT	LR, RFA, PEI	CIK cells	Loco-regional therapy alone	Adjuvant immunotherapy with CIK cells significantly increased both RFS and OS
	[80]	2016	Wang	844 HCC patients	Meta-analysis	LR, RFA, PEI	CIK cells	Loco-regional therapy alone	Adjuvant immunotherapy with CIK cells significantly increased both RFS and OS. The effect is significant only in the first 3 years from treatment
	[81]	2017	Mo	1861 HCC patients	Meta-analysis	LR, RFA, PEI	AIT	Loco-regional therapy alone	Adjuvant AIT lowers risk of mortality and tumor recurrence
	[82]	2018	Zhao	964 HCC patients	Meta-analysis	LR, RFA, PEI	AIT	Loco-regional therapy alone	Adjuvant AIT decrease the early recurrence and mortality of postoperative HCC
	[83]	2019	Lee	162 HCC patients with extended follow-up for 60 months	Multicenter RCT	LR, RFA, PEI	CIK cells	Loco-regional therapy alone	Adjuvant immunotherapy with CIK cells significantly increased both RFS and OS

AIT: Adoptive immunotherapy; ATVAC: ex vivo activated T-cell transfer; BCLC: Barcelona Clinic Liver Cancer; BSC: best supportive care; CIK: cytokine-induced killer; DCs: dendritic cells; HCC: hepatocellular carcinoma; LR: liver resection; IL: interleukin; MVI: microvascular invasion; NS: not specified; PEI: percutaneous ethanol injection; PSM Propensity score matching; RCT: randomized clinical trial; RFA: radiofrequency ablation; RFS: recurrence-free survival; R0: edges of the resected specimen free of disease; TKI: tyrosine kinase inhibitor (sorafenib, lenvatinib, apatinib); TTP: time to progression; TTR: time to recurrence.

#### 4.2.2. Immunotherapy-Based Adjuvant Treatments

Overall, data available on adjuvant therapy for HCC with TKIs seem to indicate that treatments targeting only angiogenesis could be ineffective and could benefit from the association with molecules having complementary mechanism of action. In fact, in addition to the well-known risk factors of relapse (e.g., high tumor burden, microvascular invasion), numerous immune mechanisms have been shown to play a role in HCC recurrence. Interestingly, the presence of low-grade tumor infiltration by immune effector cells (e.g., CD4+ T cells, CD8+ T cells and NK cells) is associated with a greater risk of recurrence compared to HCC with higher levels of immune infiltration [84,85]. For these reasons, immunotherapy was evaluated in the adjuvant therapy setting of HCC.

In this regard, some studies have analyzed the role of cancer vaccination (Table 2). Through the activation of effector T-cells and the development of an immunological memory, this vaccine should alter the tumor microenvironment and induce tumor-specific immunological effects, potentially favoring an adjuvant action after HCC treatment [27]. The first data provided by Kuang et al. [73] through a small phase II randomized trial demonstrated the safety, feasibility and efficacy of adjuvant autologous formalin-fixed tumor vaccine for preventing postsurgical recurrence of HCC. Compared to the group of patients not receiving adjuvant therapy, the risk of recurrence in vaccinated patients was reduced by 81%, with a significant prolongation of time to first recurrence and improvement in both RFS and OS. More recently, Lee et al. [75] evaluated the role of adjuvant infusion of autologous dendritic cells (DCs) pulsed with tumor-associated antigens after loco-regional treatment for HCC. DC vaccine significantly reduced the risk of recurrence of non-RFA group patients, whereas it unexpectedly increased this risk in RFA group. Finally, Shimizu et al. [74] analyzed the role of adjuvant combination therapy of both DC vaccines and CD3-activated T-cell transfer (ATVAC) after LR for HCC. Adjuvant combination significantly improved RFS and OS (24.5 and 97.7 months in the adjuvant ATVAC group and 12.6 and 41.0 months in the LR alone group, respectively).

Some clinical trials evaluated the efficacy of adjuvant immunotherapy using CIK or LAK cells (adoptive immunotherapy, AIT) after loco-regional treatment for HCC, with not univocal but overall favorable results (Table 2). Before others, Takayama et al. [76] evaluated the safety and efficacy of infusion of autologous lymphocytes activated with recombinant interleukin-2 and antibody to CD3 in patients with HCC undergoing curative LR. They showed that the adjuvant AIT decreased the recurrence rate by 18% and significantly prolonged the time to first recurrence compared with LR alone. However, no impact was demonstrated on the OS. Similar results (recurrence rate reduction, RFS prolongation, no impact on OS) were later confirmed also with adjuvant immunotherapy with CIK cells by Hui [77] and Xu [78] et al. In contrast, the RCT by Lee et al. [79] and the subsequent 5-year follow-up [83] showed the efficacy of adjuvant therapy with autologous CIK cells in determining a significant increase in both RFS and OS. In particular, RFS rate was 44.8% in the immunotherapy group and 33.1% in the control group after 5 years of follow-up, with significantly reduced risk of recurrence (HR: 0.67; 95% CI: 0.48–0.94) and death from all causes (HR: 0.33; 95% CI: 0.15–0.76) for both. Overall, all the meta-analyses available to date [80,81,82] agree in confirming the efficacy of adjuvant AIT in increasing both RFS and OS significantly. However, this benefit appears to be time-dependent as it lasts only the first 3 [80,82] or 5 years [81] after treatment. AIT would, in fact, be able to effectively counteract the occult intra- or extrahepatic neoplastic dissemination responsible for early recurrences but would not affect the carcinogenic substrate determined by cirrhosis, which is the cause of de novo tumorigenesis and late recurrence, instead [27].

In the context of immunotherapeutic drugs, ICIs could radically change the scenario of adjuvant therapy in the treatment of HCC. In addition to lowering the risk of early recurrence by improving systemic clearance of occult residual disease, unlike TKIs and AIT, adjuvant therapy with ICIs could also have a chemo-preventive effect as it reduces the incidence of de novo HCC by activating immune surveillance processes [86]. This could also decrease the late recurrence rate, overcoming the limitations showed by previous systemic therapies. Although the rationale for the use of ICIs in adjuvant therapy protocols is extremely promising, no data on this issue are available yet. However, there are several ongoing clinical trials aiming to evaluate the efficacy of ICIs in the setting of adjuvant therapy after loco-regional treatment for HCC. IMbrave 050 (NCT04102098) is one of the most interesting, randomized, open-label phase III trial, launched at the beginning of 2020 with the purpose to evaluate the effect of dual PD-L1/VEGF blockade using atezolizumab plus bevacizumab in high-risk HCC after curative resection or ablation [87]. The first results are expected by the end of 2023. Similarly, phase III double-blinded, two-arm KEYNOTE-937 (NCT03867084) [88] and CheckMate 9DX (NCT03383458) [89] trials are two ongoing studies with the aim to assess the safety and efficacy, respectively, of pembrolizumab (the former) and nivolumab (the latter) versus placebo as adjuvant therapy in HCC patients with complete radiological response after LR or RFA. Finally, EMERALD-2 (NCT03847428) is a phase III, randomized, double-blind, placebo-controlled, multi-center trial aiming to assess the efficacy and safety of durvalumab in combination with bevacizumab or durvalumab monotherapy or placebo as adjuvant therapy in HCC patients at high risk of recurrence [90]. It is likely that patients at high risk of recurrence (high tumor burden, large or multifocal neoplasm, poor degree of differentiation, microvascular invasion) could enjoy the greatest benefit from adjuvant therapy with ICIs [91]. The results of these and other trials could provide useful data for the validation of adjuvant immunotherapy schemes after loco-regional treatment and define the predictors of treatment response, opening the way for a new era in the management of HCC patients together with selecting those who could benefit more from these therapeutic protocols.

#### 4.2.3. Impact of Other Therapies on the Risk of HCC Recurrence

Although it cannot be considered an antineoplastic therapy, the antiviral treatment for chronic HBV infection in patients with HCC deserves special mention. In fact, the presence of viral replication correlates with an increased risk of both early and late recurrence. Patients undergoing LR for HCC with high preoperative HBV-DNA levels show lower median OS and RFS compared to those with low viral load [5,92]. If the association between active HBV infection and increased risk of late recurrence appears intuitive in relation to the persistence of the cause of liver damage and hepatocarcinogenesis, the higher risk of early recurrence seems to be related to the greater probability (about 40% more) of microvascular invasion in viraemic versus non-viraemic patients [93]. In the presence of both high [93,94] and low [95] pre-operative viral load, starting antiviral therapy after loco-regional treatment (LR or RFA) allows to reduce both early and late HCC recurrence rates [96] and improve OS [97]. Despite comparable efficacy on virological outcomes, tenofovir disoproxil fumarate (TDF) therapy seems associated with a significantly lower risk of recurrence after LR compared to entecavir (ETV) [98]. Indeed, 5-year HCC recurrence rate after LR is 33.6% in patients treated with TDF and 44.5% in those treated with ETV [98]. Unlike ETV, TDF induces the synthesis of high serum levels of interferon-lambda 3 (IFN-λ3) [99], which is able to exert a strong antitumor activity [100,101]. The antineoplastic action of TDF-induced IFN-λ3 seems to be additive to the ability to stop viral replication and turn off liver necroinflammatory activity, which is common to both ETV and TDF. No difference in recurrence rates between the two drugs was noted after RFA, instead [102].

The same concepts also seem applicable for treatment with direct-acting antiviral agents (DAAs) during chronic HCV infection. After an initial phase in which some data had suggested potential increased risk of early recurrence after HCV eradication using DAAs [103,104], numerous studies confirm that recurrence rates after antiviral therapy did not differ between patients who received IFN-based or IFN-free therapy [105,106]. On the contrary, the most recent data suggest lower risk of early and late HCC recurrence after viral eradication achieved by DAA [107,108], as well as an increase in OS [108,109] and improvement in hepatic [110] and extrahepatic [111,112] outcomes. Some predictors, including the baseline liver stiffness, could allow to stratify the residual risk of HCC occurrence or recurrence [113].

Other non-chemotherapy drugs were finally evaluated for their impact on HCC recurrence rates after loco-regional treatment. In this regard, some retrospective data would indicate that the use of angiotensin II receptor 1 blockers (sartans) after RFA is associated with longer OS and delayed time to recurrence [114]. However, specific clinical trials appear necessary in order to give preferential indication for the use of this class of drugs in the treatment of comorbidities (e.g., arterial hypertension) [115].

## 5. Future Perspectives

**Figure 1:**
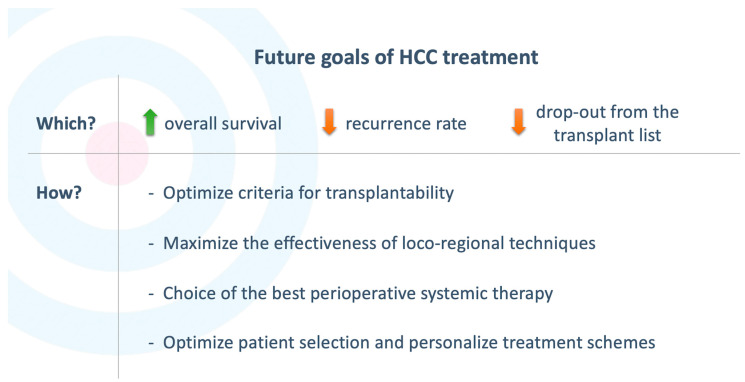
Goals and key points in optimizing HCC treatment.

Compared to the status quo, the treatment of HCC should aim at obtaining a significant improvement in OS rates, a reduction in recurrence rate and, in patients scheduled for liver transplantation, a reduction in the risk of drop-out (Figure 1). For this purpose, the optimization of transplantability criteria and the effectiveness of loco-regional techniques is certainly essential. Furthermore, the lack of effective systemic drugs and the standardization (rather than customization) of treatments have made combined therapeutic approaches unsuccessful up to now [45]. The current availability of effective drugs and the continuous development of new molecules require a significant re-evaluation of current therapeutic schemes. In particular, it appears increasingly clear that an effective systemic therapy must involve the concomitant use of multiple complementary and synergistic drugs. In this regard, the most promising scenario concerns the combination of anti-angiogenic drugs and PD-1/PD-L1 axis inhibitor drugs. 

In addition to enhancing the efficacy of systemic therapies in advanced HCC, such combinations could also allow the use of adjuvant and/or neoadjuvant therapies in the HCC setting, reducing the current high recurrence rates after curative loco-regional therapy. 

Beyond the development of effective drugs, in the future scenario, the selection of patients likely to benefit most from neoadjuvant or adjuvant therapy and the identification of the most appropriate therapeutic schemes will also be fundamental (Figure 1) [116]. In the past years, several models have been developed in order to predict the risk of recurrence in patients with HCC and establish the ideal treatment [91]. These models were mainly based on tumor burden (size, number of lesions, macrovascular invasion, location, extrahepatic metastases), histological grade, hepatic functional reserve and patient performance status. Though relevant, these data do not currently guarantee a personalization of the treatment. In fact, similar clinical pictures show frequently different behaviors and responses to treatment. An attempt to personalize the treatment could use data relating to post-procedural biochemical and radiological behavior, which in some cases has been shown to be predictive of response. For example, post-TACE transient transaminase elevation has been shown to be predictive of objective response to treatment [117]. Therefore, patients who do not experience adequate post-procedural cytolysis may be candidates for adjuvant therapy schemes. Moreover, similarly to other neoplasms (e.g., breast or lung), the molecular biology of the tumor could further guide the personalization of treatment. In fact, the availability of drugs (e.g., ICIs) able to act on different molecular pathways (and not through a generic cytotoxic effect) provides a rationale for customization of therapeutic schemes and drugs to be used in the individual case.

Once the predictive biomarkers for response to treatment have been identified, both pre- and post-operative histological and immunohistochemical evaluation of the tumor will probably be crucial, highlighting the importance of biopsy, typically not required for HCC diagnosis. In this regard, studies on in vivo models have suggested that the expression of PD-1 on CD8+ T-cells could be related to a favorable response to PD-1/PD-L1 inhibitors [118]. Similarly, high levels of FGFR4 have been associated with a better response to the lenvatinib plus anti-PD-1 combination therapy and could be used to identify patients who would benefit most from this therapy [33]. Furthermore, HCC with high tissue expression of VEGF and/or FGF shows a significantly superior objective response to lenvatinib treatment compared to neoplasms with low or intermediate VEGF and FGF expression [119]. Conversely, the best response to treatment with sorafenib was found in cases of HCC with intermediate expression levels of VEGF and FGF ligands. In this setting, an interesting proteomic study evaluating surgically resected primary tumor tissues from early-stage HCC was recently performed by Jiang et al. [120]. The authors identified patterns of protein signatures and pathways that are altered in proteomic subtypes of HCC. Proteomic alterations that identify drug-targetable proteins may provide a powerful tool for selecting patients with HCC subtypes associated with poor prognosis and/or poor response to therapy, and who might benefit from further targeted treatment.

Predictive biomarkers of response to treatment (e.g., markers of angiogenic activity) can be found not only in the cancer cell or in tumor microenvironment but also in the serum. However, the identification of specific systemic biomarkers able to provide biomolecular information of the tumor such as to predict in a rapid and non-invasive way its susceptibility to treatment is still at the dawn in the setting of liver tumors. This method, currently called “liquid biopsy”, is already being used in various types of non-hepatic neoplasms (e.g., colorectal, lung), but to date, it still finds no space in the diagnosis and treatment of HCC. However, some preliminary data are already available. For example, it is known that serum AFP levels correlate with the risk of early recurrence [5]. AFP values > 400 ng/mL have found to be associated with an adequate response to treatment with ramucirumab, a VEGF-2 inhibitor [121]. In this setting, Finn et al. [119] evaluated the correlations between serum or tissue biomarkers and outcomes from REFLECT trial. The authors highlighted that higher baseline fibroblast growth factor 21 (FGF-21) levels could be predictive for longer OS in patients treated with lenvatinib compared with those treated with sorafenib.

As mentioned previously, CD8 T cells can be responsible for a response against several cancer types. Pre-existing T-cell infiltration and PD-L1 expression can be positive prognostic factors in a variety of malignancies [122]. In patients with advanced melanoma, the ratio of Tex-cell (T effector cells) reinvigoration after PD-1 blockade to tumor burden can predict response to treatment with PD-1 inhibitors [123]. In particular, it has been noted that even a robust reinvigoration of Tex-cells from anti-PD-1 therapy could be clinically ineffective if the tumor burden is high. Therefore, in cases of melanoma, the ratio of Tex-cell reinvigoration to tumor burden, PD-L1 expression and mutational phenotype can be used as predictive markers of response to anti-PD-1 treatment. Furthermore, in melanoma patients, the low expression of Batf3 (+) DCs in pretreatment tumor biopsies was associated with an increased risk of recurrence after neoadjuvant ipilimumab + nivolumab [124]. Whether these results can also be extended to the HCC setting is currently unknown. Preliminary data suggest that in patients with HCC, baseline serum interleukin-15 was significantly correlated with RFS prolongation in patients receiving DC immunotherapy [75].

Finally, the analysis of circulating tumor DNA (ctDNA) can provide important genetic information of tumor cells and have a potential diagnostic and prognostic value. Interestingly, He et al. [125] analyzed the mutations on plasma ctDNA in 50 tumor-associated genes in patients with HCC, showing that the mutation rate of TP53, RET, FGFR3 and APC were significantly higher in patients with multiple tumors or with metastasis than in patients with single tumor. Although these data need to be validated, they may indicate that patients with high mutant allele frequency in these genes may benefit more from neoadjuvant therapy [28,125].

The predictive role of other serum biomarkers (lens culinaris agglutinin-reactive AFP, des-γ-carboxy prothrombin, proteins induced by vitamin K absence/antagonist-II) is currently under evaluation [28]. Therefore, similarly to other neoplasms, the study and identification of predictors of systemic treatment response must also be researched in the HCC setting.

## 6. Conclusions

In patients who are not candidates for liver transplantation, loco-regional treatments for HCC are burdened by unacceptable recurrence rates, especially early ones, and by poor long-term prognosis. Occult intrahepatic metastases are present in nearly half of patients with liver cancer. Perioperative combination therapies to prevent recurrence after curative liver resection or ablation is a critical issue for improving long-term survival of HCC patients. Neoadjuvant treatment schemes could allow to re-stage the neoplasm, to increase the number of patients eligible for loco-regional treatments and to improve its radicality. Adjuvant treatment protocols could instead favor a significant reduction in recurrence rate and consequent improvement in OS. Therefore, in patients who are not eligible for LT, the most effective systemic treatment schemes could include both a neoadjuvant and an adjuvant phase. Although conclusive data are not yet available, immunotherapy and, in particular, immune checkpoint inhibitors seem to have the most suitable characteristics to mark an epochal turning point in the treatment of HCC, especially if combined with anti-angiogenic therapies. In this regard, they have already demonstrated superiority in the treatment of advanced HCC compared to TKIs and are now used in combination treatments for non-hepatic neoplasms. Ongoing trials will provide answers to currently unanswered questions. If validated, these treatment schemes will need adequate patient selection in order to maximize results. However, the main obstacle to adequate patients’ selection is represented by the lack of predictive biomarkers on tissue or serum. Further studies are necessary not only to define the efficacy of adjuvant and/or neoadjuvant schemes but also to identify performing predictive factors of response to treatment that will guide us increasingly towards a precision medicine. We are, perhaps, at the dawn of a new era.
